# How Positive Affect Modulates Proactive Control: Reduced Usage of Informative Cues Under Positive Affect with Low Arousal

**DOI:** 10.3389/fpsyg.2012.00265

**Published:** 2012-07-27

**Authors:** Kerstin Fröber, Gesine Dreisbach

**Affiliations:** ^1^Department of Psychology, University of RegensburgRegensburg, Germany

**Keywords:** cognitive control, positive affect, arousal

## Abstract

An example of proactive control is the usage of informative cues to prepare for an upcoming task. Here the authors will present data from a series of three experiments, showing that positive affect along with low arousal reduces proactive control in form of a reduced reliance on informative cues. In three affect groups, neutral or positive affective picture stimuli with low and high arousal preceded every trial. In Experiments 1 and 2, using a simple response cueing paradigm with informative cues (66% cue validity), a reduced cue validity effect (CVE) was found under positive affect with low arousal. To test the robustness of the effect and to see whether reactive control is also modulated by positive affect, Experiment 3 used a cued task switching paradigm with predicitive cues (75% cue validity). As expected, a reduced CVE was again found specifically in the positive affect condition with low arousal, but only for task repetitions. Furthermore, there was no difference in switch costs between affect groups (with and without task cues). Taken together, the reduced CVE indicates that positive affect with low arousal reduces proactive control, while comparable switch costs suggest that there is no influence of positive affect on reactive control.

## Introduction

Studying issues of cognitive control is of major interest for the understanding of human cognition and action. The dual mechanisms of control (DMC) framework by Braver and colleagues (Braver et al., 2007; Braver, 2012) suggests that cognitive control operates in two distinct modes, proactive control and reactive control. Reactive control is assumed to be transiently activated in a just-in-time manner as soon as a high interference event is detected. In contrast, proactive control is supposed to be activated by the anticipation of upcoming interference. So, reactive control serves as a “late correction” mechanism to solve interference after its onset, whereas proactive control serves to prevent interference before it occurs. For this purpose, goal-relevant information is actively sustained in preparation for an optimized behavior in the upcoming cognitively demanding event. The DMC framework further claims that successful cognition relies on the variability of these two cognitive control functions, and that various factors – intrapersonal, interpersonal, or situational – can lead to a bias in favor of one mode of control strategy over the other. An example for such a biasing factor is affect.

Dreisbach (2006), for example, investigated affective modulations of cognitive control with an AX Continuous Performance Task (AX-CPT). The author could show that specifically positive affect and not negative affect – manipulated via pictures from the International Affective Picture System (IAPS, Lang et al., 1999) preceding every trial – leads to a more flexible but also less stable behavior. In the AX-CPT participants have to press a prespecified target response key to the target “X” but only if it follows the cue “A.” If X follows another letter (e.g., B) or A is followed by another letter than X (e.g., Y), the non-target response key has to be pressed. Critically, the cue A is highly informative about the occurrence of X (70% frequency of AX trials, whereas the other trial types BX, AY, and BY occur with 10% frequency each), therefore it can be assumed that in this task there is a strong bias in favor of a proactive control strategy with active maintenance of the cue information to optimize performance. Likewise, the cue B is also very informative, as it unequivocally predicts a non-target response. Dreisbach (2006) found improved performance in AY trials, but worsened performance in BX and BY trials under positive affect. This result was interpreted as evidence for a reduced maintenance of the cue, because subjects in the positive group showed costs when a to be maintained goal had to be executed (BX and BY trials; less stability) and benefits when a to be maintained goal unexpectedly changed (AY trials; more flexibility). According to the DMC framework (Braver et al., 2007; Braver, 2012), these results might provide evidence that proactive control is reduced under positive affect, because there is less usage of the cue to prepare the upcoming task (see also Compton et al., 2004). The increased flexibility, as indicated by the better performance on AY trials under positive affect, however, might as well be interpreted in terms of increased reactive control. In line with this interpretation, a recent study (van Wouwe et al., 2011) – also using the AX-CPT, but manipulating affect via emotional film clips before the actual experiment – more directly addressed the question whether positive affect influences proactive or reactive control by including measures of event related potentials (ERP). In line with the Dreisbach (2006) study, they found improved behavioral performance in AY trials, that is, on trials on which a cue-induced response tendency has to be overcome. However, unlike the previous study, the authors did not find impairment in BX and BY trials, where the cue unequivocally announced the non-target response. Based on these behavioral results and the supporting ERP data, van Wouwe et al. (2011) concluded that cue usage, and hence proactive control, did not differ between their positive and neutral group but that, instead, reactive control as soon as the target stimulus appeared was enhanced under positive affect. Considering these mixed results so far, one aim of the present study is to further clarify whether positive affect modulates proactive or reactive control. One obvious difference between both studies might be the specific mood induction procedure (namely, IAPS pictures vs. film clips). Related to that, it is conceivable that different arousal levels in the positive affect groups of both studies might account for the different results as arousal is an inherent and variable but often neglected feature of affect (Russell, 1980; Posner et al., 2005). Furthermore, arousal differences might help to explain the mixed results found in the literature so far. To our knowledge, there is only one study (Vogt et al., 2008) that shows that highly arousing affective stimuli increase the cue validity effect (CVE) of informative cues. This study, however, investigated attentional allocation to affective stimuli as it used these affective stimuli as cues in a spatial cueing task. Therefore, that study showed that highly arousing affective stimuli can attract and bind attention, but it could not answer whether or not affective arousal influences the reliance on neutral informative cues. Thus, another aim of the present study was to explore the role of arousal on positive affect effects, when affect is not confounded with the cues.

In sum, following the results of the previous study from our lab (Dreisbach, 2006) we wanted to show that positive affect with similar (low) arousal levels as used before reduces proactive control in form of a reduced usage of informative cues. This positive affect group (positive_low_ hereafter) was compared to a neutral control group and another positive affect group with higher arousal levels (positive_high_ hereafter). In Experiments 1 and 2, we used a spatial response cueing task with spatially congruent target response mappings. A bias in favor of a proactive control strategy was induced by using informative cues, that is, the probability of validly cued trials was more than 50% but less than 100%. In this response cueing task, a peripheral, informative cue indicated a possible target location and thereby primed the congruent response. Furthermore, the higher probability of valid cues (66%) should promote the usage of a proactive control strategy resulting in a reliable CVE, that is, faster responses and fewer errors in validly cued trials. A reduction of proactive control should consequently reduce the CVE, because less usage of the cues would minimize the benefits in validly cued trials as well as the costs in invalidly cued trials.

For more direct evidence that specifically proactive control and not reactive control is influenced by positive affect, Experiment 3 used a task switching paradigm. Comparing task switching performance with and without informative task cues enabled the investigation of affective influences on reactive control (as measured by switch costs) and proactive control (as measured by the CVE) in a single experiment.

## Experiment 1

Following previous results (Compton et al., 2004; Dreisbach, 2006) we expected to find a decrease in proactive control in form of a reduced CVE in the positive_low_ group as compared to the neutral group in the response cueing task with informative cues of Experiment 1. Because arousal differences were not considered in previous studies^1^ or were confounded with the cues (Vogt et al., 2008) we had no *a priori* expectations concerning different outcomes in the positive_low_ and positive_high_ group.

### Method

#### Participants

Sixty-six undergraduate students of Regensburg University participated in the experiment for course credit or 5 Euro. Sixty-two subjects (see Results for exclusion criteria) were included into the final data analysis (Mean age = 24.13 years, SD = 3.95, range = 20–38, 53 female). Participants were assigned randomly to the three affect groups (19 neutral, 21 positive_low_, 22 positive_high_). All participants signed informed consent and were debriefed after the session.

#### Apparatus and stimuli

A computer with a 17″-monitor (display resolution at 1024 × 768 pixel), running E-Prime 2.0 (Psychology Software Tools, Sharpsburg, USA) was used for experiment presentation and data acquisition. Viewing distance was held constant at 50 cm by using a chin rest. Responses were collected via a QWERTZ-keyboard, with the y- and m-key serving as left and right response keys.

To be able to manipulate valence and arousal independently we used pictures from the IAPS (Lang et al., 1999) as affect induction procedure. These pictures are known to reliably elicit specific affective reactions even with short presentation durations (Codispoti et al., 2009), and the elicited emotional reactions maintain and even sensitize – but do not habituate – with repetitive exposure to pictures of the same valence (Bradley et al., 1996; Smith et al., 2005). For each affect condition we chose 10 pictures: The neutral picture set had medium valence levels (*M* = 4.99), and low arousal levels (*M* = 2.45), whereas both positive picture sets were high in valence (*M* positive_low_ = 7.99; *M* positive_high_ = 7.25) but differed in arousal levels (*M* positive_low_ = 4.55; *M* positive_high_ = 6.30). Neutral pictures included household objects like plates or cups, positive_low_ pictures showed babies and families, and in the positive_high_ group sport and adventure pictures were displayed. It should be noted, that no erotica were used in the positive_high_ group to prevent different gender influences, and because erotica seem to be a special category with effects differing from non-sexual positive, highly arousing pictures (Most et al., 2007). All pictures were presented in landscape format and color, adjusted to a size of 800 × 600 pixel, and positioned centered on a gray background.

The fixation cross, cue and target were all displayed in black ink and bold on gray background. The fixation cross was presented at the center of the screen in font size 32 pt. The target (a single dot) and the cue (the “§”-symbol) appeared 8.64 cm to the left or right of the fixation cross in font size 55 pt.

#### Procedure

Each trial started with the presentation of the fixation cross for 500 ms, followed by an IAPS picture for 350 ms. After another short fixation period (200 ms) the cue was presented left or right of the fixation cross for 200 ms. The target appeared after a variable inter stimulus interval of 50 or 150 ms, which was included to reduce premature responses to the cue, and remained visible until the participant pressed the spatially congruent response key. Participants were instructed to react as fast as possible while avoiding errors. In case of an error, the German word for error (“Fehler”) was presented for 1000 ms as feedback.

To assure that all participants started with a similar mood, all participants passed a 5-min relaxation exercise – comprised of relaxing music and spoken instructions for muscle relaxation – prior to the actual experiment. These instructions were standardized mp3-files presented via stereo headphones. Subsequently, 12 practice trials without IAPS pictures enabled the participants to get used to the cueing task. These practice trials were followed by two experimental blocks, in which an IAPS picture preceded every trial. Both blocks consisted of 120 trials (80 valid and 40 invalid), separated by a short break. The trial procedure within each block was pseudo-random: Each block consisted of 10 sequences of 12 trials and within these 12 trials the only constraint was that cues and targets appeared equally often on the left and the right side. Affective pictures were drawn from the set of the picture pool at random without replacement until all pictures had been presented once and then the procedure started all over again.

#### Design

A 3 (affect: neutral vs. positive_low_ vs. positive_high_) × 2 (Cue validity: valid vs. invalid) mixed factors design was used. Affect was manipulated between, and Cue validity varied within participants.

### Results

#### Data analysis

The practice trials as well as the first trial of each experimental block were excluded from analyses. In addition, error trials, trials following an error, and trials with reaction times (RT) below 150 ms or above 1500 ms were excluded (4.31% of the data). Furthermore, RTs differing more than 3 SD from individual means were considered as outliers and also removed prior analysis (1.21% of the trials). The data of two participants were excluded from further analyses, because of too many errors (individual mean error rates 11 and 14% while overall error rate was 2.23%). Another two subjects had to be excluded due to untypical RTs throughout the experiment. One was exceptionally slow (*M* = 492 ms) in comparison to mean RTs of his affect group (*M* positive_low_ = 344 ms), and the other participant got continuously slower throughout the experiment and also had high mean RTs (*M* = 411 ms, while *M* neutral = 349 ms). Of the remaining data, mean RTs and error rates of each design cell (see Table 1) were entered into a 3 (Affect: neutral vs. positive_low_ vs. positive_high_) × 2 (Cue validity: valid vs. invalid) mixed factors analysis of variance (ANOVA).^2^

**Table 1:** **Mean RTs (in ms) and error rates (in %) in the spatial response cueing task of experiment 1 as a function of Affect group and Cue validity**.

	Affect group					
	Neutral		Positive_low_		Positive_high_	
	Valid	Invalid	Valid	Invalid	Valid	Invalid
RT (SD)	332 (21.4)	367 (35.5)	332 (33.3)	357 (40.69)	320 (26.0)	363 (43.9)
Errors (SD)	0.24 (0.38)	4.11 (3.1)	0.19 (0.3)	3.05 (3.16)	0.09 (0.23)	5.64 (3.95)

#### Error data, overall analysis

The overall ANOVA for the error data brought up a main effect of Cue validity, *F*(1, 59) = 90.35, *p* < 0.001, $\eta_{\text{p}}^{\text{2}}=0.605$. Fewer errors were made in valid than invalid trials (0.17 vs. 4.27%). The main effect Affect, *F*(2, 59) = 2.68, *p* = 0.077, $\eta_{\text{p}}^{2}=0.083$, did not prove reliable. But we found a significant interaction of Affect × Cue validity, *F*(2, 59) = 3.45, *p* < 0.05, $\eta_{\text{p}}^{\text{2}}\text{ = 0.105}$. Planned comparisons showed a reduced CVE in the positive_low_ group compared to the positive_high_ group (*F* = 6.73, *p* < 0.05). The CVE in the neutral group was descriptively between both positive groups, but did not differ significantly from either group (*F*s < 2.51, *p*s > 0.118). The overall error rate was 2.23% (SD = 1.85).

#### RT data, overall analysis

**Figure 1:**
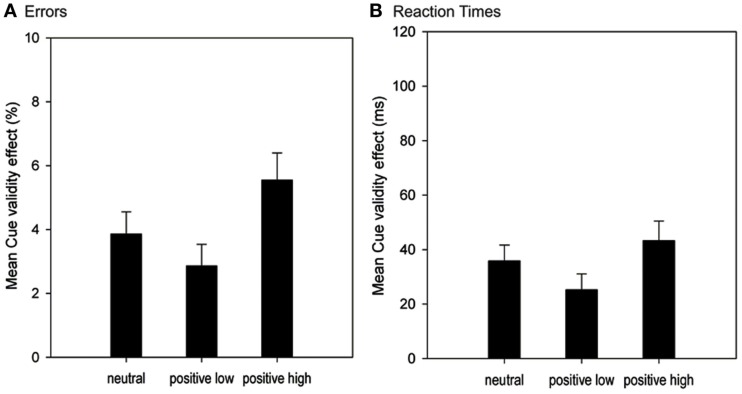
**Mean cue validity effects (CVE) in the spatial response cueing task of Experiment 1 as a function of Affect group**. The **(A)** represents CVE differences in error rates (in %), the **(B)** represents CVE differences in RTs (in ms). Error bars represent 1 standard error of the mean.

We found a significant main effect of Cue validity, *F*(1, 59) = 88.86, *p* < 0.001, $\eta_{\text{p}}^{\text{2}}\text{ = 0}\text{.601}$. Participants responded significantly faster after valid than after invalid trials (328 vs. 363 ms), resulting in an overall CVE of 35 ms. The main effect of affect as well as the interaction of Affect × Cue validity did not prove reliable (all *F* < 2.08, all *p* > 0.133). Even though we did not find a significant interaction of Affect × Cue validity in the RT analysis, the descriptive data resembles the results found in error rates (see Figure 1). CVE was smallest in the positive_low_ group (25 ms), intermediate in the neutral group (35 ms), and largest in the positive_high_ group (43 ms). Because the neutral group was more of a descriptive baseline – it differed on both valance and arousal levels from the positive groups – we conducted an additional analysis without the neutral group to search more directly for a possible arousal effect on proactive control.

#### Arousal effect, positive_low_ vs. positive_high_

A 2 (Arousal: positive_low_ vs. positive_high_) × 2 (Cue validity: valid vs. invalid) mixed factors ANOVA revealed a significant main effect of Cue validity, *F*(1, 41) = 54.19, *p* < 0.001, $\eta_{\text{p}}^{\text{2}}\text{ = 0}\text{.569}$. Participants responded faster after valid trials (326 vs. 360 ms), resulting in a CVE of 34 ms. The interaction of Arousal × Cue validity, *F*(1, 41) = 3.74, *p* = 0.059, $\eta_{\text{p}}^{\text{2}}\text{ = 0}\text{.084}$, was on the threshold of significance. Therefore, we additionally calculated the JZS-Bayes factor (Rouder et al., 2009), which gives information about the probability of a hypothesis conditionally on observed data. JZS-Bayes factor was 0.895, which means that there is indeed some evidence in favor of a difference in CVEs between positive_low_ and positive_high_ group. The main effect of Arousal did not prove reliable (*F* < 1, *p* = 0.787).

### Discussion

Experiment 1 resulted in preliminary evidence for a reduction of proactive control under positive affect. The positive_low_ group had the smallest CVE, an effect that was significant in the error data and just at the threshold of significance in the RT data. Interestingly, the CVE was increased in the positive_high_ group indicating an increase of proactive control under positive affect with high arousal. However, there were only descriptive but no statistically significant differences between the neutral group and either positive group. Experiment 2 was run to collect more empirical support for the modulation of the CVE by positive affect with differing arousal levels.

## Experiment 2

We used a very simple response cueing task in Experiment 1, which resulted in very fast overall RTs (*M* = 345 ms) and a low overall error rate (2.23%). Therefore, marginally significant differences between groups might be due to a floor effect. To increase variance and thereby provide room for affective modulations, we increased task difficulty in Experiment 2. To assure that both experiments were still comparable we used the same cueing task with informative cues (66% Cue validity) in combination with a concurrent math task. Based on the results of Experiment 1 we expected to find a reduced CVE in the positive_low_ group, but an increased CVE in the positive_high_ group, compared to the neutral group.

### Method

#### Participants

Another 60 students of Regensburg University participated in the experiment for course credit or 5 Euro. Fifty-five subjects (see Results for exclusion criteria) were included into the final data analysis (Mean age = 22.86 years, SD = 3.79, range = 19–45, 40 female). Participants were assigned randomly to the three Affect groups (18 neutral, 19 positive_low_, 18 positive_high_). All participants signed informed consent and were debriefed after the session.

#### Apparatus and stimuli

Apparatus and stimuli were the same as in Experiment 1 except for the numbers presented in the math task. The numbers 1–5 were presented centrally, in black ink and in size 32 pt. Responses in the math task had to be typed in with the number keys of the first row of the keyboard.

#### Procedure

Procedure in Experiment 2 was the same as in Experiment 1 with the following exceptions: First, in each trial of the cueing task the first fixation was replaced by random numbers 1–5 for 800 ms. These numbers were part of the additional math task. Participants performed the cueing task, and at the same time had to add up the random numbers. Every 12 trials subjects were asked to type in the result of the summation task, which was followed by an informative feedback (3500 ms). Second, the actual experiment was preceded by a math test to assure that the Affect groups did not differ according to their calculating skills. To this end, we used a subtest of the Leistungsprüfsystem (a German IQ-test; L-P-S, Horn, 1983), which requires adding up lines of 10 random numbers from 2 to 9 under speeded conditions and is therefore similar to the actual experimental situation. And third, because of the increased task difficulty we added additional practice blocks. The first block comprised 12 trials of the spatial response cueing task. The next practice block (24 trials) introduced the math task in addition to the response cueing task. It included two complete math task cycles of 12 trials with feedback. In a final practice block (12 trials) an IAPS picture preceded every cueing trial. Data acquisition took part in the following three experimental blocks with 120 trials each (80 valid and 40 invalid trials, 10 math task cycles per block).

#### Design

A 3 (Affect: neutral vs. positive_low_ vs. positive_high_) × 2 (Cue validity: valid vs. invalid) mixed factors design was used. Affect was manipulated between, whereas Cue validity varied within participants.

### Results

#### Data analysis

We checked for group differences in calculating skills before the experiment and during the experiment with an ANOVA on performance in the L-P-S subtest as well as in the additional math task. For analysis of error rates and RTs in the cueing task, trials with math task responses differing more than two from the correct result were excluded from analysis (6.31% of the data).^3^ Further preprocessing was the same as in Experiment 1, which resulted in the exclusion of another 6.83% of the trials. Furthermore one participant of the neutral group was excluded because he did not follow the instructions. Also two subjects of the positive_low_ group had to be excluded. The first made too many errors in the math task (76.7%, while mean error rate was 14.7%), and the second made too many errors in the cueing task (14.8%, while mean error rate was 1.3%). Finally, two participants of the positive_high_ group were excluded from further analysis, because they were exceptionally slow (715 and 894 ms, while mean RTs were 448 ms). Of the remaining data, mean RTs and error rates of each design cell (see Table 2) were entered in to a 3 (Affect: neutral vs. positive_low_ vs. positive_high_) × 2 (Cue validity: valid vs. invalid) mixed factors ANOVA.

**Table 2:** **Mean RTs (in ms) and error rates (in %) in the spatial response cueing task of experiment 2 as a function of Affect group and Cue validity**.

	Affect group					
	Neutral		Positive_low_		Positive_high_	
	Valid	Invalid	Valid	Invalid	Valid	Invalid
RT (SD)	405 (77.6)	477 (105.8)	445 (111.9)	487 (111.2)	401 (60.3)	471 (90.7)
Errors (SD)	0.21 (0.33)	3.32 (3.61)	0.09 (0.18)	1.86 (2.38)	0.0 (0.0)	2.35 (2.84)

#### Math performance

There were no differences in the performance in the L-P-S subtest between Affect groups before the experiment, *F*(2, 52) = 2.62, *p* = 0.082, $\eta_{\text{p}}^{\text{2}}\text{ = 0}\text{.092}$. Also, no significant differences between the three Affect groups were found in the additional math task during the experiment (*F* < 1, *p* = 0.395).

#### Error data, overall analysis

The overall error rate was 1.3% (SD = 1.5), and individual mean error rates were below 7.5% for all subjects. The overall ANOVA for the error data brought up a main effect of Cue validity, *F*(1, 52) = 36.63, *p* < 0.001, $\eta_{\text{p}}^{\text{2}}\text{ = 0}\text{.413}$, with fewer errors in valid than in invalid trials (0.10 vs. 2.51%). The main effect affect as well as the interaction of Affect × Cue validity did not prove reliable (*F*s < 1.37, *p*s > 0.263).

#### RT data, overall analysis

**Figure 2:**
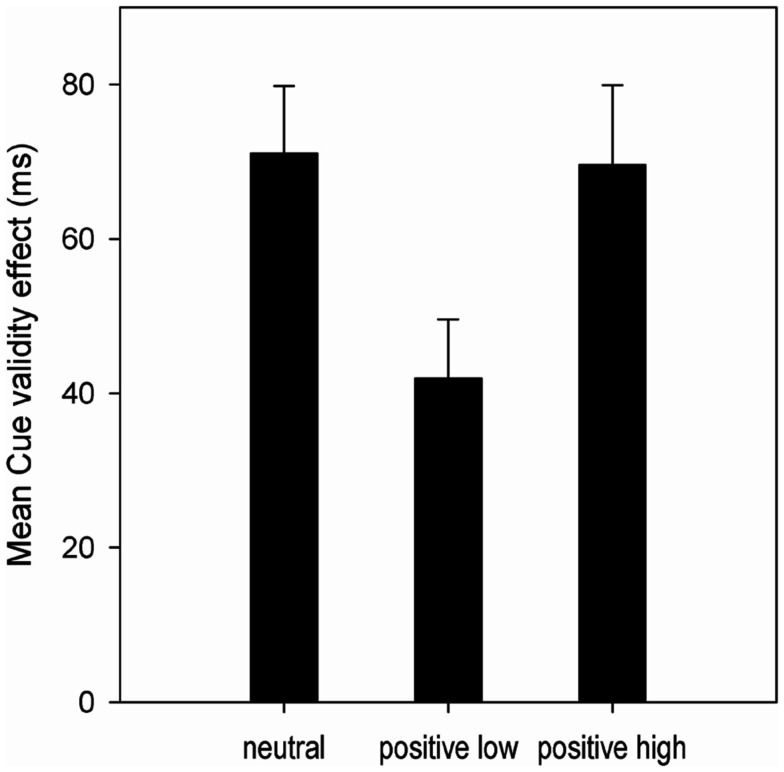
**Mean Cue validity effects (in ms) in the spatial response cueing task of Experiment 2 as a function of Affect group**. Error bars represent 1 standard error of the mean.

The ANOVA yielded a significant main effect of Cue validity, *F*(1, 52) = 142.39, *p* < 0.001, $\eta_{\text{p}}^{\text{2}}\text{ = 0}\text{.732}$. Participants responded significantly faster after valid than after invalid trials (418 vs. 478 ms), resulting in an overall CVE of 60 ms. More importantly, we found a significant interaction of Affect × Cue validity, *F*(2, 52) = 3.51, *p* < 0.05, $\eta_{p}^{\text{2}}\text{ = 0}\text{.119}$, which is depicted in Figure 2. Planned comparisons showed a reduced CVE in the positive_low_ group (41 ms) as compared to the neutral group (72 ms; *F* = 5.49, *p* < 0.05) and the positive_high_ group (70 ms; *F* = 4.94, *p* < 0.05). There was no significant difference between neutral group and positive_high_ group (*F* < 1, *p* = 0.904). Also, the main effect of Affect was not significant (*F* < 1, *p* = 0.578).

### Discussion

An increase in mean RTs from Experiment 1 to 2 (345 vs. 437 ms) indicates that we succeeded in increasing task difficulty. With this adapted paradigm we found clear-cut evidence of a reduced CVE in the positive_low_ group compared to the neutral and the positive_high_ group. This suggests that specifically positive affect with low arousal reduces proactive control in form of a reduced reliance on informative cues. In contrast to a proactive control strategy, participants in the positive_low_ group show behavioral costs in expected events (valid trials) and benefits in unexpected events (invalid trials). A problem in our simple cueing paradigm is, however, that we cannot completely rule out that the reduced CVE might also be a sign of increased *reactive* control: participants in the positive_low_ group might have used the cues just as the other affect groups but they might have been better able to overcome the pre-activated response in invalidly cued trials. This would be in line with the results by van Wouwe et al. (2011) that showed an enhancement in reactive control but no influence of positive affect on proactive control. To rule out this alternative explanation, we conducted an additional control experiment, using again a response cueing paradigm but this time employing non-informative cues.^4^ With this modification, participants could not optimize their performance with a proactive control strategy. Again, we found a significant CVE, *F*(1, 56) = 474.0, *p* < 0.001, $\eta_{\text{p}}^{2}\text{ = 0}\text{.894}$, but *no* affective modulation thereof (*F* = 1.13, *p* = 0.330). JZS-Bayes factors for CVE comparisons between the affect groups (neutral, positive_low_, and positive_high_) ranged from 1.66 to 3.75, which means the null hypothesis – no difference in CVE – was indeed more likely. So, in sum the results of the response cueing experiments speak in favor of an affective modulation of proactive control only, with a reduced reliance on informative cues under positive affect with low arousal. However, it would be even better proof, if we could show that the affective modulation of the CVE is restricted to proactive control and is not present for reactive control in a unique experiment. Therefore, we conducted Experiment 3.

## Experiment 3

The main aim of Experiment 3 was to gather more direct evidence that specifically proactive control and not reactive control is influenced by positive affect. Furthermore, we wanted to know, whether the affective modulation of proactive control can also be found for task cues (instead of response cues, as was the case with the response cueing paradigm used here and the AX-CPT in previous studies). To address these issues we employed a task switching paradigm. Task switching (for recent reviews, see, e.g., Kiesel et al., 2010; Vandierendonck et al., 2010) with univalent stimuli (e.g., digits and letters) is well suited to investigate reactive control in form of differences in switch costs. Using univalent stimuli (a given stimulus is only associated with one of the two possible tasks) and no precues, variations in switch costs can be taken as a direct indicator for reactive control processes. Furthermore, it has been shown that participants are generally very sensitive to probability cues (i.e., informative, but not 100% valid) in task switching (Dreisbach et al., 2002; Hübner et al., 2004; Miniussi et al., 2005; Dreisbach and Haider, 2006; Went et al., under review). Therefore, a cued task switching paradigm with valid and invalid cues allows not only the investigation of reactive control but also proactive control in form of differences in the CVE (like in Experiments 1 and 2). Thus, in Experiment 3 we used a task switching paradigm with a digit and a letter task that started without task cues. After the first experimental block without precues, informative task cues with a Cue validity of 75% preceded each trial. If positive affect with low arousal reduces proactive control – as Experiments 1 and 2 suggest – we should again find a reduced CVE. If positive affect, however, increases reactive control we should find a reduction of switch costs – especially so in blocks without precues.

### Method

#### Participants

Sixty undergraduate students from the Regensburg University (age *M* = 22.53 years, SD = 4.02, range = 18–36, 53 female) participated in the experiment for course credit or 5 Euro. Participants were assigned randomly to the three affect groups (20 positive_low_, 20 positive_high_, 20 neutral). All participants signed informed consent and were debriefed after the session. Because we were interested in a possible modulation of the switch costs, participants with negative switch costs were excluded and replaced (two in the neutral, three in the positive_low_, and two in the positive_high_ group).

#### Apparatus and stimuli

Apparatus was the same as in Experiments 1 and 2. Also the same IAPS picture sets were used for the three affect groups.

Eight digits (1, 2, 3, 4, 6, 7, 8, and 9) written in green and eight letters (A, E, O, U, C, K, G, and T) written in purple served as target stimuli and were presented at the center of the screen in font size 52. The color coding of the digit and letter task was counter balanced across participants. Odd numbers and vowels were always assigned to one response key, even numbers and consonants to the other, while response mapping to the left and right response key (y- and m-key on a QWERTZ-keyboard) was also counterbalanced between participants. In experimental blocks 2–4, a color coded fixation cross (purple or green) served as informative task cue.

#### Procedure

The experiment comprised one task switching block without task cues followed by three blocks including informative task cues. In the first block each trial started with an IAPS picture (350 ms) followed by a blank screen (150 ms) and a black fixation cross (1000 ms). Then the target stimulus appeared and remained on screen until the participant responded. Subjects had to decide whether a number was odd or even (digit task) or whether a letter was a vowel or consonant (letter task). Participants were instructed to react as fast as possible while avoiding errors. Feedback was given for errors only (2000 ms), each trial ended with an intertrial interval of 500 ms. Procedure in the following blocks with informative task cues was the same as in the first block except that the fixation cross was now color coded and served as a task cue for the following task. In valid trials (75% of all trials) the colored fixation cross was followed by a target stimulus in the same color, thereby enabling the preparation of the upcoming task in a proactive manner. In contrast, in invalid trials (25% of all trials) the fixation color incorrectly predicted the upcoming target color, and can therefore mislead to prepare the wrong task.

The experiment started with the same relaxation exercise that was used before in Experiments 1 and 2. Subsequently, 16 practice trials (random presentation of all target stimuli) without IAPS pictures enabled the participants to get used to the task switching procedure. This practice block was followed by 64 trials with an IAPS picture preceding every trial. Data acquisition took place in the following four experimental blocks – the first without informative task cues – with 128 trials each. Each block contained 64 digit tasks (4 × 8 numbers) and 64 letter tasks (4 × 8 letters). Stimulus presentation was pseudo-randomized with the following constraints: repeat and switch trials were evenly distributed. Immediate repetitions of target stimuli or IAPS pictures were not allowed. Task cues (96 valid, 32 invalid) were counterbalanced across all trial types.

#### Design

A 3 (Affect: neutral vs. positive_low_ vs. positive_high_) × 2 (Trial type: repeat vs. switch) design with affect as between and Trial type as within factor was used in the first block without task cues. The experimental blocks including informative task cues had a 3 (Affect) × 3 (Block: 2 vs. 3 vs. 4) × 2 (Trial type) × 2 (Cue validity: valid vs. invalid) repeated measures design.

### Results

#### Data analysis

Practice trials as well as the first trial of each experimental block were excluded from analyses. In addition, error trials, trials following an error, and trials with RTs differing more than 3 SD from individual means were also removed prior analysis (9.34% of all trials). Separate analyses were conducted for task switching performance (mean error rates and RTs) in the first experimental block without task cues and for performance in experimental blocks 2–4 with informative task cues.

#### Task switching performance, block 1 without task cues

Mean RTs (see Table 3) were entered into a 3 (Affect: neutral vs. positive_low_ vs. positive_high_) × 2 (Trial type: repeat vs. switch) mixed factors ANOVA. We found a significant main effect of Trial type, *F*(1, 57) = 106.45, *p* < 0.001, $\eta_{\text{p}}^{\text{2}}\text{ = 0}\text{.651}$, with faster responses in repeat trials (655 vs. 733 ms). The main effect of Affect as well as the interaction of Affect × Trial type did not prove reliable (all *F* < 1.97, all *p* > 0.150). The same analysis for mean error rates (see Table 3) also resulted in a significant main effect of Trial type, *F*(1, 57) = 26.82, *p* < 0.001, $\eta_{\text{p}}^{\text{2}}\text{ = 0}\text{.319}$, with less errors in repeat trials (2.28 vs. 5.97%). Again, no significant Affect effects were found (all *F* < 1.19, all *p* > 0.31). JZS-Bayes factors for differences in switch costs between the Affect groups ranged from 2.95 to 4.04, which means that it is more likely that there are indeed equal switch costs in all three groups.

**Table 3:** **Mean RTs (in ms) and error rates (in %) in the in the first experimental block of experiment 3 (task switching without task cues) as a function of Affect group and Trial type**.

	Affect group					
	Neutral		Positive_low_		Positive_high_	
	Repeat	Switch	Repeat	Switch	Repeat	Switch
RT (SD)	646 (76.9)	731 (116.7)	705 (170.5)	774 (202.0)	615 (96.9)	693 (133.5)
Errors (SD)	2.7 (2.89)	5.89 (4.68)	1.52 (2.09)	4.83 (3.72)	2.64 (2.73)	7.18 (8.19)

#### Task switching performance, blocks 2–4 with informative task cues

To check the effectiveness of the cues over time, we conducted a 3 (Affect: neutral vs. positive_low_ vs. positive_high_) × 3 (Block: 2 vs. 3 vs. 4) × 2 (Trial type: repeat vs. switch) × 2 (Cue validity: valid vs. invalid) mixed factors ANOVA for the three experimental blocks with informative task cues (see Tables 4 and 5 for mean RTs and error rates). The analysis of mean error rates resulted in significant main effects of Block, *F*(2,114) = 8.65, *p* < 0.001, $\eta_{\text{p}}^{\text{2}}\text{ = 0}\text{.072}$, Trial type, *F*(1, 57) = 37.34, *p* < 0.001, $\eta_{\text{p}}^{\text{2}}\text{ = 0}\text{.397}$, and Cue validity, *F*(1, 57) = 4.40, *p* < 0.05, $\eta_{\text{p}}^{\text{2}}\text{ = 0}\text{.072}$, as well as an interaction of Trial type × Cue validity, *F*(1, 57) = 4.19, *p* < 0.05, $\eta_{\text{p}}^{\text{2}}\text{ = 0}\text{.069}$. Planned comparisons showed significantly more errors in Block 2 (3.61%) as compared to Block 3 (2,81%, *F*(1, 57) = 7.97, *p* < 0.01) and Block 4 (2.42%, *F*(1, 57) = 14.20, *p* < 0.001). Blocks 3 and 4 did not differ significantly (*F* = 2.01, *p* = 0.162). Cue validity had no significant influence an error rates in task repetitions (2.09 vs. 2.12%, *F* < 1, *p* = 0.915), but there was a significant negative CVE in task switches (*F*(1,57) = 6.41, *p* < 0.05) with more errors in valid trials (4.30 vs. 3.27%). The interaction of Block and Trial type did not prove reliable (*F* = 2.82, *p* = 0.064). There was no significant main effect of Affect or significant interactions with affect (all *F* < 1.68, all *p* > 0.185). In the RT analysis we found significant main effects for Block, *F*(2, 114) = 19.83, *p* < 0.001, $\eta_{\text{p}}^{\text{2}}\text{ = 0}\text{.258}$, Trial type, *F*(1, 57) = 98.88, *p* < 0.001, $\eta_{\text{p}}^{\text{2}}\text{ = 0}\text{.634}$, and Cue validity, *F*(1, 57) = 19.53, *p* < 0.001, $\eta_{\text{p}}^{\text{2}}=0.255$, which were further qualified by a significant three-way interaction of these factors, *F*(2, 114) = 11.28, *p* < 0.001, $\eta_{\text{p}}^{\text{2}}\text{ = 0}\text{.165}$. Planned comparisons showed a significant interaction of Trial type × Cue validity specifically in the first block with informative task cues, *F*(1, 57) = 2.54, *p* < 0.001 (Blocks 3 and 4: all *F* < 0.07, all *p* > 0.41). Further analysis of Block 2 showed a significant CVE with faster RTs after valid cues in repeat trials (590 vs. 644 ms, *F*(1, 57) = 32.28, *p* < 0.001), but not in switch trials (667 vs. 659 ms, *F* = 1.32, *p* = 0.26). So, there was a strong cueing effect only in the first block with informative task cues, and specifically in repeat trials. The main effect of Affect as well as all other interactions did not prove reliable (all *F* < 3.36, all *p* > 0.067). With respect to our hypotheses, also in these blocks with informative task cues the Affect groups did not differ significantly in switch costs (*M* neutral = 54 ms, *M* positive_low_ = 57 ms, *M* positive_high_ = 44 ms). JZS-Bayes factors for single comparisons of switch costs ranged from 2.46 to 4.24, which further supports that switch costs were indeed comparable in all three groups. Since we were interested in the affective modulation of the CVE, we reran the analysis, this time only including Block 2 (i.e., the first block with informative task cues), the only block where the CVE was significant.

**Table 4:** **Mean RTs (in ms, SD in parentheses) in experimental blocks 2–4 of Experiment 3 (task switching with informative task cues) as a function of Affect group, Trial type, and Cue validity**.

Cue	Affect group					
	Neutral		Positive_low_		Positive_high_	
	Repeat	Switch	Repeat	Switch	Repeat	Switch
**BLOCK 2**						
Valid	568 (81.1)	639 (133.9)	588 (61.1)	661 (83.4)	615 (119.9)	702 (142.4)
Invalid	613 (111.9)	626 (92.3)	617 (103.2)	665 (92.9)	702 (176.9)	685 (125.63)
**BLOCK 3**						
Valid	561 (73.4)	617 (110.2)	590 (91.9)	648 (105.5)	600 (99.8)	665 (141.0)
Invalid	558 (82.7)	643 (151.9)	600 (104.3)	654 (125.5)	603 (123.3)	656 (124.0)
**BLOCK 4**						
Valid	557 (86.8)	595 (108.1)	566 (71.4)	608 (140.0)	591 (102.5)	635 (133.8)
Invalid	567 (109.2)	631 (151.1)	579 (81.2)	645 (140.0)	602 (115.6)	631 (126.9)

**Table 5:** **Mean error rates (in %, SD in parentheses) in experimental blocks 2–4 of experiment 3 (task switching with informative task cues) as a function of Affect group, Trial type, and Cue validity**.

Cue	Affect group					
	Neutral		Positive_low_		Positive_high_	
	Repeat	Switch	Repeat	Switch	Repeat	Switch
**BLOCK 2**						
Valid	2.76 (2.3)	5.55 (4.7)	2.27 (2.0)	4.89 (4.6)	1.87 (2.1)	4.88 (4.0)
Invalid	2.29 (3.7)	5.67 (4.8)	2.5 (4.8)	3.17 (4.6)	2.5 (5.5)	5.01 (5.3)
**BLOCK 3**						
Valid	2.27 (2.8)	4.62 (5.6)	2.39 (3.4)	4.44 (4.6)	1.67 (2.8)	4.15 (3.9)
Invalid	1.91 (3.2)	2.96 (4.2)	2.15 (3.3)	2.28 (4.0)	2.15 (3.9)	2.73 (5.2)
**BLOCK 4**						
Valid	1.7 (2.3)	4.2 (3.8)	2.39 (2.7)	2.8 (2.5)	1.52 (2.0)	3.2 (3.3)
Invalid	2.95 (3.6)	1.96 (3.2)	0.59 (2.6)	2.49 (4.2)	2.06 (4.4)	3.21 (4.9)

#### Affect effects, first task switching block with informative task cues only

**Figure 3:**
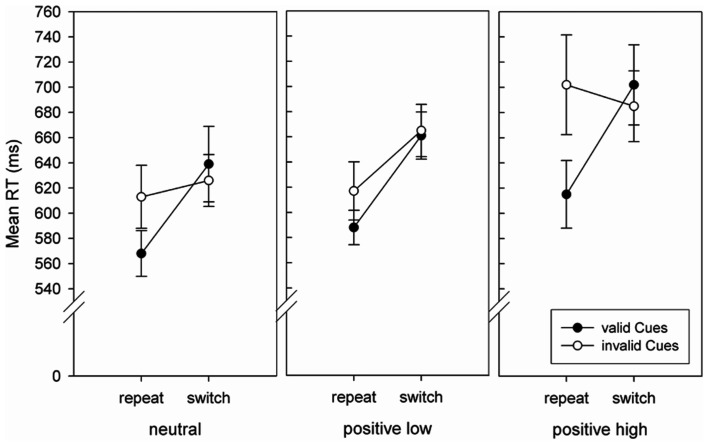
**Mean RTs (in ms) in the first task switching block with informative task cues of Experiment 3 as a function of Affect group, Trial type, and Cue validity**. Error bars represent 1 standard error of the mean.

A 3 (Affect: neutral vs. positive_low_ vs. positive_high_) × 2 (Trial type: repeat vs. switch) × 2 (Cue validity: valid vs. invalid) mixed factors ANOVA revealed significant main effects for Trial type, *F*(1, 57) = 39.46, *p* < 0.001, $\eta_{\text{p}}^{\text{2}}\text{ = 0}\text{.409}$, and Cue validity, *F*(1, 57) = 18.07, *p* < 0.001, $\eta_{\text{p}}^{\text{2}}\text{ = 0}\text{.241}$. Participants responded faster in repeat trials (617 vs. 663 ms) as well as in valid trials (629 vs. 651 ms). Furthermore, we found a significant interaction of Trial type × Cue validity, *F*(1, 57) = 22.54, *p* < 0.001, $\eta_{\text{p}}^{\text{2}}\text{ = 0}\text{.283}$. Planned comparisons showed a significant CVE in repeat trials (590 vs. 644 ms, *F*(1, 57) = 32.28, *p* < 0.001), but not in switch trials (667 vs. 659 ms, *F* = 1.32, *p* = 0.26). Most important with respect to our hypothesis, there was a significant interaction of Affect × Trial type × Cue validity, *F*(2, 57) = 3.08, *p* = 0.05, $\eta_{\text{p}}^{\text{2}}\text{ = 0}\text{.098}$, which is depicted in Figure 3. CVE was significantly smaller in the positive_low_ compared to the positive_high_ group (29 vs. 87 ms, *F*(1, 57) = 6.32, *p* < 0.05). The CVE in the neutral group (45 ms) was descriptively between both positive groups but did not differ significantly from either group (*F*s < 3.35, *p*s > 0.072). The main effect Affect and all other interactions did not prove reliable (all *F* < 1.94, all *p* > 0.15). The same analysis for mean error rates resulted only in a significant main effect of Trial type, *F*(1, 57) = 25.06, *p* < 0.001, $\eta_{\text{p}}^{\text{2}}\text{ = 0}\text{.306}$, with less errors in repeat trials (2.36 vs. 4.86%). No further significant main effects or interactions were found (all *F* < 1, all *p* > 0.47).

### Discussion

In Experiment 3 switch costs did not differ between affect groups, neither in the first experimental block without task cues nor in the following blocks with informative cues. Strong cueing effects were found only in the first block with informative task cues and specifically in repeat trials. In this block we also found an affect effect similar to the results of Experiments 1 and 2: the CVE in repeat trials was reduced in the positive_low_ group as compared to the positive high group, while the CVE was descriptively in between both positive groups in the neutral group. It is not surprising that we found an affective modulation only in Block 2, because block wise analysis of all three blocks including cues showed that the informative task cues only had an impact on performance while they were new, whereas their influence diminished with more practice in the task (RTs and error rates declined throughout the experiment, see Tables 4 and 5). The generally reduced reliance on cues over blocks might be due to the fact that the task cues were neither necessary (because univalent stimuli were used) nor entirely useful (e.g., Sudevan and Taylor, 1987). The fact that the CVE is restricted to repeat trials only was also found by Miniussi et al. (2005), and might be a consequence of anticipatory backward inhibition (Mayr and Keele, 2000; Hübner et al., 2003; Li and Dupuis, 2008): in task switching, backward inhibition refers to the phenomenon that preparation for a task switch leads to inhibition of the just executed task set, and is hence also a form of proactive control. There is plenty of evidence that the foreknowledge about an upcoming task switch suffices to trigger the inhibition of the preceding task (Mayr and Keele, 2000, Experiment 5; Hübner et al., 2003; Li and Dupuis, 2008; Went et al., under review). Applied to our data, an invalidly cued repetition already caused inhibition of the previous task resulting in performance costs when this very task unexpectedly repeats. In invalidly cued switches, on the other hand, the cue predicts a repetition and as such does not trigger backward inhibition resulting in typical switch costs – like in validly cued switches. In sum, Experiment 3 succeeded in showing that specifically proactive control and not reactive control is modulated by positive affect: Switch costs – as a measure of reactive control – were comparable in all three affect groups in the first block without task cues. Positive affect along with high or low arousal did neither improve nor impair the adaption to a (unexpected) task switch. In contrast, the CVE – as a measure of proactive control – was again modulated by affect, and indicated a reduction of proactive control in the positive_low_ group.

Together with results from Experiments 1 and 2, we thus found converging evidence that performance under positive affect with low arousal is less dependent on informative cues, indicating a reduction in proactive control. Positive affect with high arousal, on the other hand, seems to increase the usage of informative cues.

## General Discussion

Purpose of the present study was to investigate the influence of positive affect on processes of proactive control under different arousal conditions. According to the DMC framework (Braver et al., 2007; Braver, 2012) cognitive control can be divided into proactive and reactive control: proactive control means sustained preparation for an upcoming event – for example, by using informative cues to optimize performance, while reactive control means a just-in-time activation of control as soon as a demanding event appears. In three experiments with different paradigms and kinds of informative cues, we found converging evidence that positive affect with low arousal – induced via short presentation of affective pictures – reduces the CVE. These results replicate and extend previous findings (Compton et al., 2004; Dreisbach, 2006) by showing that only positive affect with low arousal but not positive affect with high arousal reduces the usage of informative cues, and by showing that these effects are not limited to response cues but can be generalized to task cues (for limitations see Discussion of Experiment 3). Furthermore, results on task switching performance in Experiment 3 strongly suggest that positive affect does *not* modulate reactive control (see also Discussion of Experiment 2 and Footnote 4) by showing that switch costs were not manipulated by affect. Taken together, the results of Experiments 1–3 support the assumption that specifically positive affect with low arousal leads to a reduction in proactive control.

In Experiment 3, we found comparable switch costs in both positive groups and the neutral group suggesting that reactive control was not modulated by affect. At first sight, this seems to be at odds with findings by Dreisbach and Goschke (2004), who found interactions of switch costs and positive affect. Their study, however, did not use a classical task switching paradigm but a cognitive set-switching paradigm. In this paradigm, participants did not have to switch between different tasks, but performed a single task only: they had to categorize a target presented in one color, while ignoring a simultaneously presented distractor in another color. Dreisbach and Goschke investigated two switching conditions of cognitive sets: After the switch, either the targets appeared in a new color, while the former target color become the distractor color (perseveration condition), or the distractors appeared in a new color, while the former distractor color became the target color (learned irrelevance condition). Positive affect diminished switch costs when switching to a new cognitive set (perseveration condition), but increased switch costs and interference by distractors in the learned irrelevance condition. Dreisbach and Goschke interpreted these very specific interactions between positive affect and switch costs as evidence for increased cognitive flexibility accompanied by costs of increased distractibility under positive affect. The task switches in Experiment 3 of this study, however, can not be differentiated by these two switching conditions. Therefore, it is no surprise that no affective modulation of switch costs was found here. However, the reduced CVE might just as well be interpreted as an index of increased cognitive flexibility. For example, Compton et al. (2004) argued that a reduced CVE can be interpreted in terms of more flexibility because the behavior is less dependent on the cue information.

**Figure 4:**
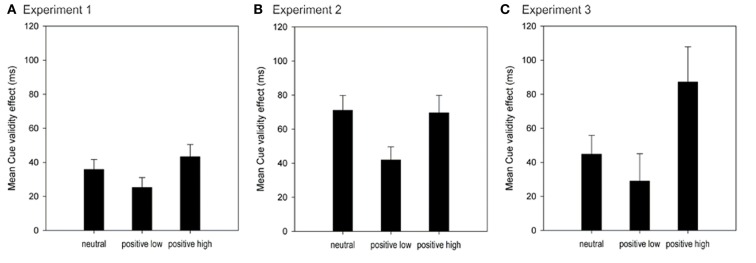
**Mean Cue validity effects (in ms) as a function of Affect group for Experiment 1 (A), Experiment 2 (B), and Experiment 3 (C)**. Error bars represent 1 standard error of the mean.

The CVE was significantly smaller in the positive_low_ compared to the positive_high_ group in both the response cueing (Experiments 1 and 2) as well as the cued task switching paradigm (Experiment 3), while the CVE in the neutral group was roughly between both positive groups (see Figure 4). But in spite of these descriptive differences between the neutral group and both positive groups, there was only once – in Experiment 2 – also a significant reduction of the CVE in the positive_low_ group compared to the neutral group (while the magnitude of the CVE was equally high in the neutral and the positive_high_ group). This lack of significant differences might be a byproduct of our procedure: each experiment started with a short relaxation exercise to create a similar baseline mood in all participants. This procedure, however, might already have resulted in a mild positive affect induction, thereby possibly reducing the differences between the neutral group and the positive group especially with low arousal. Admittedly, what speaks against this assumption is that in Experiment 2, the CVE of the neutral group actually resembled the positive_high_ group. It is, however, conceivable that the higher task demands due to the additional math task have counteracted the relaxed mood in the neutral group. Thus, the significant difference found in Experiment 2 might in fact be closer to the actual difference between neutral affect and positive affect with low arousal. Also, it can be assumed that everyday mood is generally rather mildly positive than truly neutral. Therefore, it might not be too surprising that differences between mild positive affect and neutral affect are not easily detected. However, with these constraints in mind, the observed differences in the CVE between positive affect with low arousal and neutral and positive affect with high arousal provide sufficient evidence for the conclusion that positive affect with low arousal decreases proactive control, while positive affect with high arousal seems to increase proactive control compared to neutral affect.

The reduced CVE in the positive_low_ group converges with findings from previous studies by Compton et al. (2004) and Dreisbach (2006). Compton et al. (2004) investigated associations between baseline mood state – assessed via the Profile of Mood States (McNair et al., 1971) – and performance in an attentional orienting task with informative cues. Self-reported negative affect was unrelated to attentional orienting performance. High positive affect, however, was associated with a reduced CVE, with slower responses after validly cued targets and faster responses following invalidly cued targets, compared to low positive affect. Dreisbach (2006) used the AX-CPT and found enhanced performance in AY trials, that is, in invalidly cued trials, but impaired performance in BX and BY trials, that is, in validly cued trials, under positive affect as compared to neutral or negative affect. Thus in both studies, positive affect resulted in a benefit in expected events, but also in costs in unexpected events. These findings – like our results – can be explained by a reduced usage of informative cues, which indicates a reduction in proactive control. But unlike Dreisbach (2006) a recent study by van Wouwe et al. (2011) – also using the AX-CPT – found no influence of positive affect on cue usage (no impairment in BX and BY trials), and hence proactive control, but, instead, differences between their positive and neutral group in reactive control: participants in the positive affect group showed a performance benefit and ERP differences in AY trials only, where a pre-dominant response tendency has to be overcome. In line with these results are also several studies by Kuhl and colleagues (Kuhl and Kazen, 1999; Baumann and Kuhl, 2005; Kazén and Kuhl, 2005) that used paradigms without informative cues, which means that there is not much room for proactive control. They used the Stroop task and a global-local task and found a reduction in Stroop interference and a reduced global precedence under positive affect (again no consideration of arousal differences) indicating also an enhanced ability to overcome pre-dominant response tendencies. So overall, there is evidence for increased flexibility in form of a reduction in *proactive* control (this study; Compton et al., 2004; Dreisbach, 2006), but also evidence for increased flexibility in form of a modulation of *reactive* control (Kuhl and Kazen, 1999; Baumann and Kuhl, 2005; Kazén and Kuhl, 2005; van Wouwe et al., 2011). One reason for these mixed results might be the differential affect induction procedures: the current study – like the AX-CPT study by Dreisbach – manipulated affect in a between groups design with affective pictures preceding every trial, Compton et al. investigated differences in baseline mood state, van Wouwe et al. used emotional film clips previous to the actual experiment (for a more detailed discussion on differences between the two AX-CPT studies see van Wouwe et al., 2011), and Kuhl and colleagues used a within design with random presentation of positive, negative, or neutral prime words preceding every trial. So, Compton et al. as well as van Wouwe et al. were concerned with effects of a sustained mood state – in the former case the currently existing mood state, in the latter case an induced mood state – whereas Kuhl and colleagues investigated influences of rather transient affective reactions. The affect induction procedure used in our lab (this study; Dreisbach, 2006) – affective pictures preceding every trial in a between groups design – most likely resulted in both transient and sustained affective reactions. IAPS pictures very quickly elicit typical emotional reactions with changes in cortical, autonomic, and facial activity, as well as evaluative ratings even with short presentation durations (Codispoti et al., 2001, 2009). Furthermore, repetitive exposure to pictures of the same valence leads to maintained or even sensitized affective reactions and can therefore be seen as a mood induction procedure (Bradley et al., 1996; Smith et al., 2005). For the studies reviewed here, however, the difference between sustained mood states vs. transient affective reactions does not seem to be a crucial factor to explain the different outcomes in the affective modulation of cognitive control. For example, also van Steenbergen et al. (2009) found consistent positive affect effects on the sequential modulation of response conflicts using either randomized affective signals between trials (smilies) or specific mood induction in a between groups design (van Steenbergen et al., 2010). In fact, there are other procedural factors aside from different affect induction procedures that might as well be crucial. For example, the reduced Stroop interference found by Kuhl and colleagues was restricted to conditions when intention memory is activated, that is, in the first of two consecutive Stroop tasks in a single trial (Kuhl and Kazen, 1999) or when using specific positive primes related to achievement (Kazén and Kuhl, 2005). Also, none of the above-quoted studies considered differences in arousal levels. But note that in the Dreisbach (2006) study the positive IAPS pictures had low arousal levels comparable to the ones used here. In sum, the existing literature is characterized by mixed results, which might be explained to some extent by different affect induction procedures – pictures vs. film clips vs. words, between vs. within –, differences in intention memory load, as well as different arousal levels. Therefore, future studies are clearly needed to further clarify under which conditions positive affect influences proactive or reactive control.

The fact, that we found a reliable difference in the CVE between the positive groups with low and high arousal, demonstrates that it is most important to consider both dimensions of affect – valence and arousal (cf., Russell, 1980; Posner et al., 2005). Whether there is less attention to the cues or a reduced maintenance of the cue information in the positive_low_ group cannot be answered based on behavioral results alone. But nonetheless, it remains an interesting question why positive affect in combination with low arousal reduces proactive control, whereas positive affect along with high arousal seems to increase proactive control. Reduced proactive control under positive affect with low arousal seems to converge with our everyday experience: When being in a relaxed, mildly positive mood one tends to enjoy the moment without looking ahead. This would also be in line with Carver’s (2003) coasting theory. This theory assumes a feedback function of affect: more precisely, positive affect signals better progress than necessary, and consequently reduces the effort invested in the ongoing task (=coasting). Proactive control in this sense is associated with more effort than reactive control, because it involves sustained maintenance of informative cues or task goals for an optimized behavior (Braver et al., (2007; Braver, 2012). Thus, a reduction of proactive control could be a sign of coasting: Participants in the positive_low_ group apply less effort in sustained task preparation, and instead rely on reactive control alone as soon as the target appears. This might also explain why the effects of reduced proactive control were restricted to the positive affect group with low arousal and were not found with high arousal. Obviously, coasting might not be a reasonable strategy under high arousal as any high arousal signal might rather serve as a warning or alertness signal. For example, Fuentes and Campoy (2008) showed in an attention network task that alerting tones increase the CVE, and inferred that alerting enhances the effect of informative cues. A similar explanation presents the integrative theory of locus coeruleus-norephinephrine function (LC-NE) by Aston-Jones and Cohen (2005). Arousal is associated with NE activity, and according to the integrative LC-NE theory specifically phasic LC-NE activity promotes exploitative behavior that helps to optimize task performance. Applied to our data, the short presentation of highly arousing positive pictures might have triggered phasic NE activity and thereby resulted in increased proactive control in form of a stronger usage of the informative cue, and, as a consequence, an increased CVE.^5^

## Conclusion

The DMC framework (Braver et al., 2007; Braver, 2012) assumes that there are various factors that induce a bias in favor of one type of control strategy over the other. Taken together, Experiments 1–3 resulted in converging evidence that positive affect is such a factor. Specifically, positive affect with low arousal led to a reduction in proactive control in form of a reduced reliance on informative cues. On the other hand, positive affect in combination with high arousal increased the CVE and therefore seems to promote proactive control. Reactive control, in contrast, was not influenced by positive affect.

## Conflict of Interest Statement

The authors declare that the research was conducted in the absence of any commercial or financial relationships that could be construed as a potential conflict of interest.

## Footnotes

^1^Note that in Dreisbach (2006) negative IAPS pictures were chosen to match the arousal levels of the positive affect pictures.

^2^Although previous studies (Compton et al., 2004; Dreisbach, 2006) already indicated a valence specific effect, we still included two negative affect groups – one with low and one with high arousal – in Experiment 1 to disentangle valence and arousal effects on proactive control. It turned out that negative affect in combination with high arousal led to a general increase in RT compared to the neutral group and both positive groups (*F*s > 4.13, *p*s < 0.05). Both negative groups showed greater cue validity effects than the positive_low_ group (*M* negative_high_ = 46 ms, *M* negative_low_ = 37 ms, *M* positive_low_ = 25 ms). There was no significant interaction of negative affect with low or high arousal and cue validity. The negative groups therefore contributed no information concerning the special topic of anticipation (and the special topic of this Frontiers issue). To enhance the readability of the present article results of the negative groups are therefore not presented here but can be reported on request.

^3^We wanted to be sure that participants were truly engaged with both the response cueing task and the additional math task. Therefore, we controlled for performance in the math task. But we chose a rather moderate criterion (correct response ±2) to minimize data loss, because exclusion due to math task performance meant to exclude a complete cycle of 12 cueing trials. With an absolute criterion (only correct responses included) 17.7% of all trials would have been excluded. By using the moderate criterion, we aimed to include all trials where participants genuinely tried to follow instructions.

^4^We tested 59 subjects (20 neutral, 20 positive_low_, and 19 positive_high_) in the cueing experiment with non-informative cues (50% validity). Stimuli and procedure were basically the same as in Experiment 1 except for the following changes: The fixation cross as well as the two possible target locations were always enclosed by black bordered boxes (size 100 × 100 pixels). As a cue the border of one peripheral box changed border width from 1 to 5 pt. The stimulus onset asynchrony between cue and target was changed to 50 or 150 ms, which was shorter than in Experiment 1 but equaled the inter stimulus interval of Experiment 1. Furthermore, the cue remained on screen with the target until the participant pressed a response key, and the cue validity percentage was reduced to 50%, which resulted in 60 valid and 60 invalid trials per block. All these changes were made to induce a bias in favor of a reactive control strategy. Mean error rate was 1.26% and mean RTs were 339 ms. Mean CVE were comparable in all affect groups with 40 ms in the neutral group, 43 ms in the positive_low_ group, and 35 ms in the positive_high_ group.

^5^We are aware of the fact that in the NE-LC model (Aston-Jones and Cohen, 2005) the relation between phasic NE activity and exploitative behavior vs. tonic NE activity and explorative behavior is correlational in nature and based on animal studies. That is, even if we assume that the short presentation of highly arousing picture stimuli triggers NE activity, it is still an open question, whether the activation of phasic NE activity triggers exploitative behavior in humans. Actually, the first pharmacological study addressing this issue failed to find evidence for the NE-LC involvement in human action control (Jepma et al., 2010).

## Appendix

Numbers of affective picture stimuli (Lang et al., 1999).

Neutral: 7000, 7004, 7006, 7009, 7035, 7040, 7080, 7090, 7175, 7233.
Positive_low_: 1440, 1710, 1750, 1920, 2057, 2150, 2260, 2311, 2340, 2530.
Positive_high_: 5260, 5621, 5623, 5626, 5629, 8161, 8180, 8190, 8200, 8490.
